# “Speed”: A Dataset for Human Speed Estimation

**DOI:** 10.3390/s25206335

**Published:** 2025-10-14

**Authors:** Zainab R. Bachir, Usman Tariq

**Affiliations:** Electrical Engineering Department, College of Engineering, American University of Sharjah, Sharjah P.O. Box 26666, United Arab Emirates; g00029388@alumni.aus.edu

**Keywords:** accelerometer, convolutional neural network, gyroscope, inertial measurement unit

## Abstract

Over the years, researchers have developed several speed estimation techniques using wearable inertial measurement units (IMUs). In this paper, we introduce a medium-scale dataset, containing measurements of walking/running at speeds ranging from 4.0 km/h (1.11 m/s) to 9.5 km/h (2.64 m/s) in increments of 0.5 km/h (0.14 m/s) from 33 healthy subjects wearing IMUs. We name it the “*Speed*” dataset. In summary, we present accelerometer and gyroscope data from 12 speeds and 22 subject-independent sets with the full range of 12 speeds. The data in each set consists of overlapping sections of 250 time samples (corresponding to 2.5 s, sampled at 100 Hz), and six dimensions (corresponding to the three axes of the accelerometer and three axes of the gyroscope). Each speed set contains 1775 examples. We benchmark the existing approaches used in the literature for the purpose of speed estimation on this dataset. These include support vector regression, Gaussian Process Regression, and shallow neural networks. We then design a deep Convolutional Neural Network (CNN), *SpeedNet*, for baseline results. The proposed *SpeedNet* yields an average Root Mean Square Error (RMSE) of 0.4819 km/h (0.13 m/s), following a subject-independent approach. Then, the *SpeedNet* obtained from the subject-independent approach are adapted using a portion of subject-specific data. The average RMSE for the remainder of the data for all subjects then drops down to 0.1747 km/h (0.05 m/s). The suggested *SpeedNet* yields a lower RMSE in comparison to the other approaches. In addition, we also compare the proposed method to others in terms of the average testing time, to give an idea of computational complexity. The proposed *SpeedNet*, despite being more accurate, yields real-time performance.

## 1. Introduction

The health and fitness industry has grown dramatically in the last decade. The increasing demand for fitness solutions and the advancement in electronics have made it viable to design fitness applications, watches, smart personal trainers, etc. People are becoming more interested in measuring their performance while training. Speed is one of the key performance metrics while walking/running. Many researchers have developed several speed estimation techniques using inertial measurement units (IMUs) since 1990 [1].

It is worth mentioning that the first research in human locomotion started with Eadweard Muybridge [2]. He studied a horse while running and noticed that while a horse is galloping, it will have its four legs in the air. Following Muybridge, Étienne-Jules Marey, who was interested in Muybridge’s work, decided to study the movement of flying birds and later invented a photographic rifle and eventually a chronophotograph, which helped him in his research of human locomotion. Following them came Christian Wilhelm Braune and Otto Fischer, who improved Marey’s work by using more chronophotographers and obtaining more accurate results on studying human walking [2].

Researchers have worked on many techniques for speed estimation using IMUs. These techniques are either based on the accelerometer and/or gyroscope signals or on models based on walking behavior [3]. One such technique estimates speed based on gait variable estimation [4]. Another technique is based on integrating the accelerometer data to segment a data stream into gait cycles [5,6]. In addition, some approaches employ machine learning methods such as support vector regression (SVR), Gaussian process regression (GPR), neural networks (NNs), and more recently, transformer-based models (Transformer). For instance, McGinnis et al. [7] estimate walking speed using SVR for two groups of people: healthy individuals and Multiple Sclerosis (MS) patients. The significance of this study lies in the ability of the model to predict MS patients’ speeds by training it with healthy patients’ data, which indicates a very robust model that captures the variations in walking gait for a given speed. In addition, several studies have employed GPR for speed estimation, such as in the works by Vathsangam et al. [8], Zihajehzadeh and Park [9], and Zihajehzadeh et al. [10]. The results obtained from Vathsangam et al. [8] highlight the importance of including gyroscope data for speed estimation, where it was not included in earlier studies. It also highlights the importance of including height as a feature when following a subject-independent approach. NNs were employed by researchers to predict the speed of walking by Aminian et al. [11]. However, the proposed method has limited applications to real-life scenarios as the neural network design was subject-dependent. NNs were also used by Song et al. [12]. This work employs 17 subjects, which is a fair number of participants compared to related works. The work covers a wide range of speeds, from 4.8 km/h to 15.4 km/h. However, estimating speed from an estimated stride length causes additional error in the speed estimation. Transformers have been used in several applications relevant to human pose and gait. Lu et al. [13] use transformers for gait recognition using wearable IMU sensors. Yuanshuo et al. [14] use a fusion of an RGB camera and IMU data using transformers for full-body pose estimation for rehabilitation purposes. Ahmadian et al. use transformers [15] to predict a plantar pressure image using recorded pressure points from the foot contact with the floor while walking. Note that transformers may have high memory and computational requirements, making them a less desirable choice for speed estimation in a resource-constrained device, e.g., cell phone.

Table 1 shows some of the datasets presented in the literature. While reviewing the literature, we could only find one dataset that is publicly available [16], and that is for a geriatric population walking at their preferred speeds. This encouraged us to collect a well-curated dataset and make it publicly available for the benefit of the research community.

The range of speeds and the spacing between them, along with the number of subjects, give our dataset superiority over other similar datasets, as shown in Table 1. Our *Speed* dataset has the greatest number of subjects, excluding the datasets used by McGinnis et al. [7] and Hannink et al. [16] (however, McGinnis et al. [7] have a mix of both healthy subjects and MS patients, and Hannink et al. [16] includes the geriatric population at their preferred speeds). The *Speed* dataset also has a finer range of speed steps than most, at 0.5 km/h, except for Vathsangam et al. [8] with 0.3 km/h step and even 0.16 km/h step for one subject. However, the work by Vathsangam et al. [8] uses a subject-dependent approach and only includes walking speeds. The *Speed* dataset includes both walking and running speeds; the only other case to include this is Song et al. [12], but with a larger step of 1 km/h.

The contributions of this research are twofold:

- We present a medium-scale dataset, which contains IMU measurements from 33 healthy subjects, walking/running at speeds ranging from 4.0 km/h (1.11 m/s) to 9.5 km/h (2.64 m/s) in increments of 0.5 km/h (0.14 m/s) to suit the endurance level of the participants and most healthy (not necessarily athletic) people. We name it the “*Speed*” dataset. No other dataset is available in the literature with such a diverse and fine range of speed measurements for healthy subjects. We are making the dataset publicly available under a licensing agreement. This will be a great help for researchers working in the area.
- In addition, we employ deep learning in the form of a deep CNN, for speed estimation for baseline results. CNNs have the ability to extract features from raw data and are very powerful in image recognition. Researchers have also applied them for gait variable estimation and human activity recognition [16,17]. The resulting RMSE measures show that the proposed deep CNN beats the other methods for speed estimation from the literature. We call our baseline network *SpeedNet*. We leave other improvements in the model architecture, such as incorporating attention mechanisms [18], etc., for future researchers to build upon the baseline results.

The significance of our research lies in the dataset obtained and the CNN developed. The dataset can be used by researchers to advance the research in human speed estimation, or any human gait variable-related studies. Our method of using convolutional neural networks can also be employed in future research for developing training algorithms and applications that can be deployed even on a smartphone. CNNs are better suited for any data that lies on a grid, has correlation amongst adjacent values, and can suffer from issues such as dimension hopping of data (where similar patterns appear at a different location). This is a typical case in images, where neighboring pixels are highly correlated. In addition, if you translate an image by a few pixels, although the overall resulting image may be very similar to the original one, at the local level, the information “hopped” to adjacent locations. In our case for speed estimation, CNN is a good choice as well, since we are dealing with accelerometer and gyroscope data coming from walking/running. The data is defined over a regular grid, which are sampled time values in our case. The data contains periodic motion with high correlation to adjacent samples, like images with correlation in adjacent pixels. Also, the stride formation may be shifted, like images where similar patterns may appear elsewhere in the image, due to translation, for example. In addition, our eventual goal is to implement the algorithm on a smart phone. CNNs are a good compromise in terms of performance and computation, e.g., in comparison to long short-term memory networks (LSTMs), in this regard [19]. The authors leave the study of other sequence modeling algorithms, e.g., LSTMs, transformers, etc., for other researchers, who can then use our results as a benchmark. This also includes works from other domains, e.g., natural language processing for sequence labeling, e.g., [20]. Hence, to summarize, the primary aim of this study is to collect a human locomotion dataset from healthy subjects at varying speeds, with low-cost wearable sensors. This dataset can then be used by other researchers to develop algorithms and applications built around these sensors. This can also help the fitness industry to develop specialized equipment, e.g., shoes. In addition, our other aim is to develop a model architecture that can predict human speed with wearable IMU sensors, to provide a baseline performance, and to benchmark it against some other pre-existing speed estimation algorithms. We leave other improvements in the model architecture, such as incorporating attention mechanisms (transformers) [18], etc., for future researchers to build upon these results.

## 2. Methodology

### 2.1. Data Collection

We recruited 33 healthy participants that are moderately fit and non-athletic. They do not suffer from any chronic diseases or have health problems that prevent them or put them at risk from walking/running on the treadmill at the selected speeds. They were asked to walk/run on a treadmill with various controlled speeds matching their endurance level. The subjects’ age ranges from 18 to 30 years old. The mean height of the subjects was 174.76 cm with a standard deviation of 8.68 cm. In addition, the mean body mass of the subjects was 79.95 kg, with a standard deviation of 13.43 kg. The data collection was done with approval from the Institutional Review Board (IRB) at the American University of Sharjah. All the participants signed an informed consent and were free to leave at any time without any repercussions.

**Figure 1:**
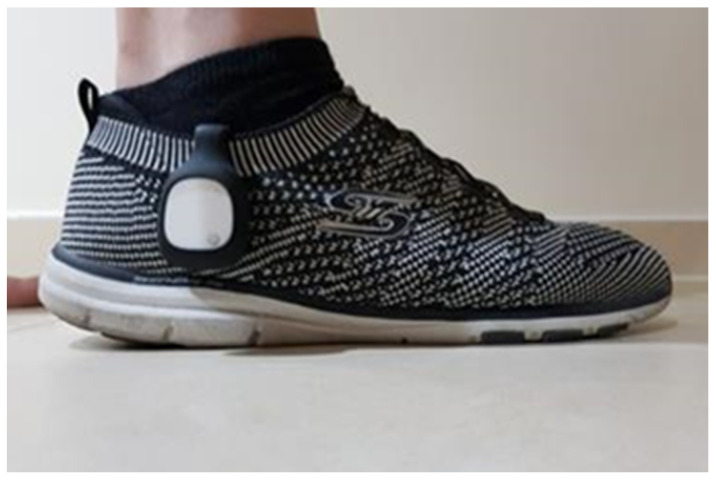
IMU sensor attachment on the outer side of the right shoe.

We recorded their movement with a small (MetaMotionR+) sensor attached to their right shoe via a clip-on case. This is shown in Figure 1.

MetamotionR+ IMUs have the following specifications:

- Accelerometer range: ±2 g, ±4 g, ±8 g, ±16 g, 16-bit resolution.
- Gyroscope range: ±125°/s, ±250°/s, ±500°/s, ±1000°/s, ±2000°/s, 16-bit resolution.
- Static orientation error < 0.5°.
- Dynamic orientation error < 2°.
- Resolution of orientation < 0.05°.

We chose the sensor location to be at the foot level, as it has been shown to be a good location for speed estimation purposes [21]. The data is sampled at 100 Hz and is streamed via Bluetooth Low Energy to the mobile phone using the MetaBase application [22]. We then retrieve the three-dimensional accelerometer and gyroscope data as an Excel sheet.

We opted for 12 speeds ranging from 1.11 m/s (4.0 km/h) up to 2.64 m/s (9.5 km/h) in a 0.14 m/s (0.5 km/h) step increments, in this research. We chose this range of speeds keeping in view the endurance level of the participants. We requested the participants to walk for the first 6 speeds and jog/run for the latter ones. We recorded each speed for a duration of 3 min to capture enough variations.

Not all of the subjects recorded their data for all of the chosen speeds. Hence, we randomly made up 22 subject-independent sets with the full range of speeds. Here, we combined the data from one, two, or three participants to give the full range of chosen speeds. The dataset is summarized in Table 2.

### 2.2. Pre-Processing

We compared various approaches, used in the literature, to the proposed *SpeedNet* to provide a baseline performance for speed estimation. *SpeedNet* is described further in the following section. The other approaches include SVR [7], GPR [9], and NN [12]. For each of these methods, we extracted the same features as did the respective researchers for a fair comparison. However, we tuned the variables of the respective algorithms with respect to our dataset, for optimal performance.

In our proposed *SpeedNet*, we fed the overlapping data segments/windows of 250 samples as input for each speed. We used this window length, keeping in view that we capture at least one stride in each window. The sampling rate was 100 Hz; hence, 250 samples correspond to 2.5 s. We used the raw accelerometer and gyroscope data, with three axes each, as features. Hence, the input feature dimensions were 6 × 250. The number of windows for each speed was 1775 with 96% overlap. Deep learning algorithms need a reasonable amount of data to train. It is well known that breaking up sequences into subsequences with overlap for training has certain advantages. These include increasing the data to train and making the CNN more robust towards misalignments. We chose the 96% overlap, although it is considered a high overlap, to produce more inputs for our training and for improving the model’s robustness. It does not impact the computational efficiency; it only creates more data for the model to train on.

### 2.3. SpeedNet

We chose a CNN design over LSTM because LSTMs are computationally expensive when compared to CNNs, and with building a mobile application in mind as a future goal, choosing CNNs over LSTMs made more sense.

We pose the speed estimation problem as a regression problem. Hence, *SpeedNet*, though based on AlexNet [23] in design, conducts regression in place of classification. We use it as the baseline algorithm for comparison with future work by other researchers. The proposed *SpeedNet* is based on seven distinct layers: input layer, convolutional layer, batch normalization layer, rectified linear unit (ReLU) layer, pooling layer, fully connected layer, and a regression layer. Some of these layers are then repeated to produce a *SpeedNet* architecture comprising 19 layers.

The structure of SpeedNet can be summarized as follows. The input layer, that is 250 × 6 in dimensions, is followed by a convolutional layer with 16, 6 × 5 filters and horizontal strides of one step each, with padding. This is followed by batch normalization and ReLU application [23]. After that, max pooling over a 1 × 2 block is performed. Then, this convolution–normalization–ReLU–max pooling block is replicated three more times with 32, 64, and 128 filters, respectively. The filter sizes in each of these blocks are 6 × 3. After the last block, we have a fully connected layer, which is then followed by a regression layer in the end. The SpeedNet is learned using a mean squared error (MSE) loss function.

**Figure 2:**
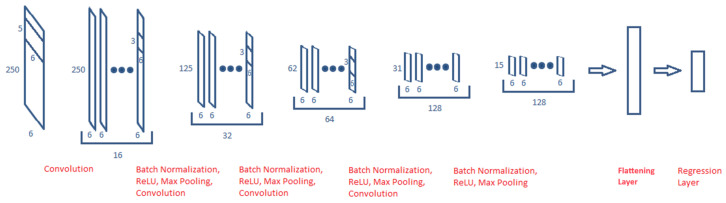
*SpeedNet* Architecture showing the 250 × 6 input as it passes through all the layers of the CNN. The smaller segment indicates the filter size applied in the convolution layer, and the number of filters increases as the input progresses into the CNN from 16 to 128. Notice the dimension for each output is decreasing by half in one dimension after applying the max pooling layer. The following are the layers’ dimensions: input layer: 250 × 6; output of first convolutional layer: 250 × 6 × 16; output of second convolutional layer: 125 × 6 × 32; output of third convolutional layer: 62 × 6 × 64; output of fourth convolutional layer: 31 × 6 × 128; output of fifth convolutional layer: 15 × 6 × 128; and output of fully connected regression layer: 1 × 1.

We decided on these variables by cross-validation. Other alternatives, such as increasing the layers, etc., did not improve the performance. For further details on specific operations in *SpeedNet*, the readers are referred to [23]. Figure 2 shows the architecture of the proposed *SpeedNet*. This provides the baseline results for a deep learning based architecture on the proposed *Speed* dataset.

### 2.4. Experimental Setup

We adopted a leave-one-out experimental protocol for all our experiments. We used 19 sets for training, 2 sets for validation (e.g., when to stop training to avoid overfitting), and 1 set for testing. The process is then repeated until all the sets have been used as testing sets. Then, we report the average RMSE over all the sets.

## 3. Results and Discussion

Table 3 lists the RMSE for each one of the 22 sets, along with the average RMSE and standard deviation, for *SpeedNet*, SVR [7], GPR [9], and NN [12]. Note that the *SpeedNet* gives the lowest average RMSE (0.4819 km/h) with the lowest standard deviation (0.2693 km/h) compared to the other approaches.

**Figure 3:**
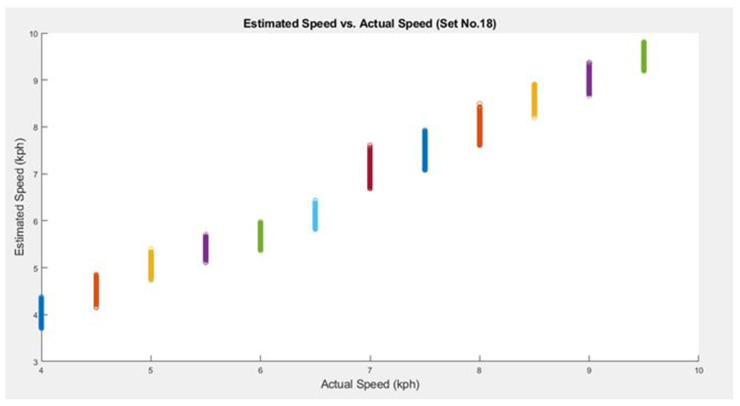
Estimated speed vs. actual speed (Set 18). The different colors represent the different ground truth (actual) speeds, as also evident from the horizontal axis.

One can notice from Table 3 that Set 18 gave the lowest RMSE of 0.1963 km/h with *SpeedNet*. We plot the estimated speeds for this set in Figure 3. From Figure 3, we find that the estimated speeds vary around the actual speeds within a small range with no under/over-estimation of the speeds. Note that we found the average correlation coefficient for *SpeedNet* to be 0.9831. This indicates a very high positive correlation between the actual speeds and estimated speeds.

**Figure 4:**
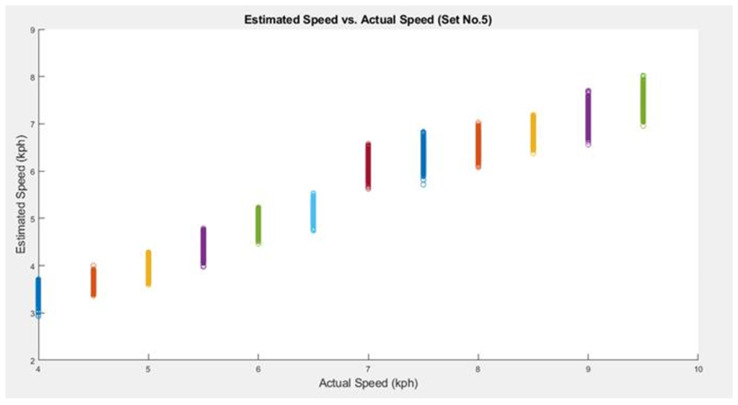
Estimated speed vs. actual speed (Set 5). The different colors represent the different ground truth (actual) speeds, as also evident from the horizontal axis.

In addition, the highest RMSE, in the case of *SpeedNet*, was for Set 5. Hence, we plot the estimated speeds for this set in Figure 4. This set consists of the data from the second-tallest subject, with a height of 191 cm, which is far from the average height of 174.8 cm. One can notice the expected effect from Figure 4, where it is very clear that the speeds are underestimated. We conjecture that if we have enough data from subjects of varying heights, this error can be reduced further. On a side note, we also experimented with height as a feature; however, it did not improve the performance. Perhaps, the *SpeedNet* architecture is extracting that implicit information from the sensory data itself.

**Figure 5:**
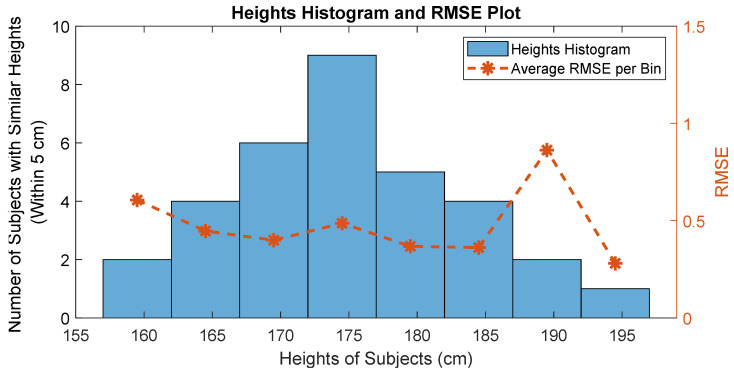
Histogram of heights of subjects along with the superimposed plot of their respective average RMSEs.

To dig deeper, we plotted a histogram of the heights of subjects, with a bin width of 5 cm. We then superimposed the plot of the average RMSE of the subjects (in the respective bins) on top of the histogram. This plot is shown in Figure 5. The plot shows that the heights of the subjects are, more or less, normally distributed. In addition, no clear relationship between the number of subjects in a bin and their respective average RMSE was observed. With the exception of a couple of bins, the average RMSE lies around a similar range. We conjecture that with more data, the RMSE exceptions will go away. We can see in the bins in which there is a slight jump (due to under- or overestimation), there are only two subjects each.

Another interesting point worth mentioning is that the average RMSE for the other methods in Table 3 was higher than what was reported earlier in the literature. There may be several reasons behind the higher RMSE from simulating the prior works in our dataset. The most important reason is perhaps that we are using a different dataset. In our dataset, the spacing between the consecutive speeds is much smaller in comparison to the other works. Our speeds are merely 0.5 km/h apart. This is important, as this makes the problem more difficult. The spacing between the speeds in the works by both McGinnis et al. [7] and Song et al. [12] is 0.9 km/h or higher. Another reason could be the smaller variation in the heights and/or the body masses of the subjects in the other works. In the work by McGinnis et al. [7], the heights range from 160 cm to 180 cm, and in the work by Zihajehzadeh and Park [9], the subjects’ body masses have a mean of 65 kg with a standard deviation of 11 kg. In the work by Song et al. [12], the subjects have a mean height of 175.4 cm with a standard deviation of 4.7 cm and a mean body mass of 62.7 kg with a standard deviation of 3.74 kg. In comparison, in our dataset, the mean height is 174.76 cm with a standard deviation of 8.68 cm and the mean body mass is 79.95 kg with a standard deviation of 13.43 kg. This again makes our problem more challenging. Note that the other datasets are not publicly available. Our dataset, on the contrary, can be requested under a licensing agreement.

**Figure 6:**
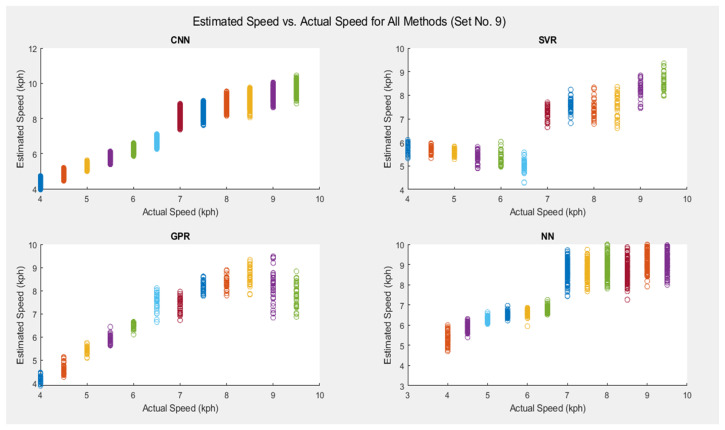
Comparison of estimated speed vs. actual speed for all methods (Set 9). The different colors represent the different ground truth (actual) speeds, as also evident from the horizontal axis.

Figure 6 shows a comparison of the estimated speeds between the four methods for Set 9. The sub-figures show that the correlation of *SpeedNet* is the strongest in comparison, with a slight overestimation of the running speeds. One can also note that the SVR performs poorly for the lower speeds. The performance is also far from optimal for the higher speeds. GPR has a wider spread overall; however, it is more particularly so for the higher speeds. In addition, the NN method performs poorly for the higher speeds.

We also compared *SpeedNet* to the other approaches in terms of testing time. This comparison was conducted for Set 9 in terms of the average running time required for the model to estimate one speed. The numbers were similar for the other sets. Table 4 shows the running time. It can be seen that GPR takes the longest time to estimate the speed, while the NN is the fastest in estimating the speed. *SpeedNet* and SVR have running times that are very close to each other. The comparison was conducted using an old laptop with an Intel i5 (8th generation) processor.

### Subject-Dependent Approach

Here, we adapt the *SpeedNets* with a small portion of testing data. The motivation is for practical reasons. It is similar to the systems where a learned model is adapted for a particular user. For example, several speech recognition systems ask users to say some phrases during their first-time use to fine-tune their variables for the user. We conducted more training iterations on the existing *SpeedNets*, obtained from the subject/set independent approach explained in the previous sections, using half of the testing set data (this was used completely for testing, in the earlier experiments). We then tested the modified network on the remaining 50% of the test set data. Here, we also followed the same leave-one-out approach as our earlier experiments. The test sets’ RMSE is shown in Table 5. One can see much lower RMSE values in comparison to Table 3.

## 4. Concluding Remarks

In conclusion, we present a large *Speed* dataset with 33 subjects for speed estimation. The dataset can be obtained from the authors under a licensing agreement. We then presented our proposed deep CNN, *SpeedNet*, which outperformed the other works in the literature. *SpeedNet* provides the baseline results for a deep learning architecture on the *Speed* dataset. We obtained an average RMSE of 0.4819 km/h (0.13 m/s) for a subject-independent approach and a much lower average RMSE of 0.1747 km/h (0.05 m/s) for the subject-dependant approach. In addition, *SpeedNet* estimates speed within 0.0526 s, which makes it suitable for real-time applications. The *Speed* dataset is publicly available under a licensing agreement. We have a large number of subjects, and the dataset includes both walking and running speeds with a small difference of 0.5 km/h (0.14 m/s) between any two consecutive speeds. In the future, we plan to include the algorithms developed in this work in a mobile application. The idea for the app is to use it for walking/running competitions on the treadmills. Almost all mobile phones have a built-in accelerometer and gyroscope that can be used as a substitute for the attached IMU in our research. However, the mobile phone placement will certainly not be in the same position as our IMU, and that will be accounted for in the future design if the attached IMU is to be replaced with the one in cell phones.

Limitations: Our design is limited to treadmill usage and might not generalize well to over-ground walking/running due to gait pattern differences, such as slower gait and shorter, consistent strides with treadmill walking/running. In addition, it might not generalize well to people with gait impairments, as the data was obtained from healthy non-athletic adults.

## Figures and Tables

**Table 1:** Dataset comparison.

Reference	Public Availability	Sensor	Sampling Frequency	# of Subjects	Healthy (H) / Patient (P)	Speeds (km/h)
*Speed* (Ours)	Publicly available	Acc + Gyro	100 Hz	33	H	4.0, 4.5, 5.0, 5.5, 6.0, 6.5, 7.0, 7.5, 8.0, 8.5, 9.0, 9.5
Bugané et al. [4]	-	Acc + Gyro	50 Hz	22	H	Preferred speed
Yang et al. [5]	-	Acc + Gyro	100 Hz	7	H	9, 9.9, 10.8, 11.7, 12.6
Chew et al. [6]	-	Acc + Gyro	128 Hz	4	H	8, 9, 10, 11
Hannink et al. [16]	Publicly available	Acc + Gyro	102.4 Hz	116	Geriatric	Preferred speed
McGinnis et al. [7]	-	Acc	50 Hz	A: 10	H	A: 1.8, 2.7, 3.6, 4.5, 5.4
				B: 37	H: 7 and MS P: 30	B: comfortable and ±20%
Vathsangam et al. [8]	-	Acc + Gyro	100 Hz	8	H	4, 4.5, 4.8, 5.3, 5.6, 6.1, 6.4
						Subject 9: 4, 4.16, 4.32, 4.48, 4.64, 4.8, 4.96, 5.12, 5.28, 5.44, 5.6, 5.76, 5.92, 6.08, 6.24, 6.4
Zihajehzadeh et al. [9]	-	Acc	100 Hz	15	H	Slow, Normal, Fast
Zihajehzadeh et al. [10]		Acc + Gyro + Mag	100 Hz	10	H	1.8, 2.7, 3.6, 4.5, 5.4, 6.3
Aminian et al. [11]	-	Acc	40 Hz	5	H	Preferred speed
Song et al. [12]	-	Acc	200 Hz	17	H	4.8, 6.8, 7.8, 9, 10.2, 11.4, 12.6, 13.8, 15, 15.4 with different inclines

**Table 2:** Summary of the “*Speed*” dataset.

Summary of the “*Speed*” Dataset	
Number of subjects	33
Number of subject-independent sets	22
Examples per subject-independent set	1775
Length of each example	250 samples
Number of speeds	12
Speeds (km/h)	4.0, 4.5, 5.0, 5.5, 6.0, 6.5, 7.0, 7.5, 8.0, 8.5, 9.0, 9.5
Sampling frequency	100 Hz
Dimensionality—gyroscope data	3
Dimensionality—accelerometer data	3

**Table 3:** RMSEs for CNN, SVR, GPR, and NN methods across all the sets, using a leave-one-out approach, along with the respective standard deviations (the values are in km/h).

Sets	*SpeedNet* RMSE	SVR RMSE	GPR RMSE	NN RMSE
1	0.7546	0.8978	0.3334	0.8656
2	0.2326	1.0531	0.4825	0.7239
3	0.2097	0.4742	0.4929	0.8333
4	0.3671	1.1569	0.5923	2.5707
5	1.3399	1.7824	0.4166	1.8838
6	0.3085	0.7745	0.4989	0.9453
7	0.2669	1.6783	0.3318	0.8213
8	0.6732	0.9335	1.7265	0.6503
9	0.5721	0.9355	1.7262	1.0527
10	0.3097	1.6199	0.6005	1.1037
11	0.6521	1.8774	1.7263	1.206
12	0.657	0.9303	0.3397	0.918
13	0.3415	1.2703	0.4543	0.7662
14	0.4967	2.0762	0.4871	0.7508
15	0.2624	0.9382	0.4908	1.0484
16	0.4489	3.8393	0.4224	0.913
17	0.6249	2.6435	0.5142	1.0394
18	0.1963	0.739	0.3993	0.8137
19	0.8042	2.3644	1.727	0.8002
20	0.3379	0.7825	0.3443	0.934
21	0.2449	1.6502	0.6994	0.9793
22	0.4995	0.7577	0.4226	1.8615
Average RMSE	**0.4819**	1.4171	0.6922	1.0673
Standard Deviation	**0.2693**	0.7939	0.5071	0.4598

**Table 4:** Average testing time (seconds) on Set 9.

CNN	SVR	GPR	NN
0.0526	0.0393	0.18	0.0034

**Table 5:** RMSE for all 22 sets (subject dependent). (RMSE is in km/h).

Sets	RMSE	Sets	RMSE
1	0.169	12	0.2205
2	0.1511	13	0.1873
3	0.1728	14	0.1493
4	0.18	15	0.178
5	0.1584	16	0.1762
6	0.1803	17	0.1521
7	0.202	18	0.1652
8	0.1701	19	0.2114
9	0.2241	20	0.1607
10	0.1539	21	0.1788
11	0.1571	22	0.1452
Average RMSE		0.1747	
Standard Deviation		0.0227	

## Data Availability

The dataset will be made available under a licensing agreement.
